# Magnetoionography enhances diagnostic accuracy of magnetocardiography in coronary artery disease

**DOI:** 10.1038/s41598-025-14054-4

**Published:** 2025-08-25

**Authors:** Dominic Dischl, Dominik D. Kranz, Sebastian Bannasch, Andrej Gapelyuk, Ulf Landmesser, Jai-Wun Park, Niels Wessel

**Affiliations:** 1 German Heart Centre Munich, TUM University Hospital, Lazarettstrasse 36, 80636 Munich, Germany; 2 Department of Neurology with Experimental Neurology, Charité – Universitätsmedizin Berlin, Charité Campus Mitte, Charitéplatz 1, 10117 Berlin, Germany; 3 Berlin Institute of Health, Luisenstraße 65, 10117 Berlin, Germany; 4 Department of Physics, Humboldt Universität zu Berlin, Robert-Koch-Platz 4, 10115 Berlin, Germany; 5 Biomagnetik Park GmbH, Forsthöhe 26, 21149 Hamburg, Germany; 6 Department of Cardiology, Angiology and Intensive Care Medicine, German Heart Center Berlin, Charité – Universitätsmedizin Berlin, Hindenburgdamm 30, 12200 Berlin, Germany; 7 Department of Human Medicine, MSB Medical School Berlin GmbH, Rüdesheimer Str. 50, 14197 Berlin, Germany

**Keywords:** Magnetocardiography, Magnetoionography, Coronary artery disease, Molecular biophysics, Cardiovascular biology, Biological physics, Diagnostic markers

## Abstract

Coronary artery disease (CAD) remains a leading cause of morbidity and mortality worldwide. Traditional diagnostic approaches, including coronary angiography and electrocardiography, have limitations in detecting ischemia and microvascular dysfunction, leading to misdiagnoses and unnecessary interventions. This study evaluates the efficacy of magnetoionography (MIG), a novel parameter extension of magnetocardiography (MCG), in improving the detection of CAD by analyzing potential intracellular cardiac currents. We conducted a prospective study including 93 CAD patients and 36 healthy controls. All CAD patients underwent non-invasive MCG measurements before coronary angiography. Conventional MCG parameters were assessed alongside retrospectively together with the MIG-derived indices, focusing on the characterization of intracellular ion currents during repolarization. MIG analysis significantly improved CAD detection accuracy. The inclusion of MIG parameters in a stepwise linear discriminant analysis increased sensitivity from 90.3% (MCG alone) to 93.5% and specificity from 76.5 to 85.3%. Key discriminative parameters included Heart Rate, Current Moment Dynamics, and Dipolarity Index for ST Segment (all stress). Our findings support the further study of MIG for its potential use in clinical practice as a non-invasive, highly sensitive diagnostic tool for CAD. Through intracellular cardiac currents captured by an MCG system, the MIG parameter extension may offer deeper pathophysiological insights, potentially enhancing risk stratification and early disease detection.

## Introduction

Coronary artery disease (CAD) stands as a predominant global health concern, causing ~ 17.9 million deaths annually^1^. Its prevalence is driven by modifiable risks—hypertension, dyslipidaemia, diabetes, smoking and physical inactivity—and non-modifiable factors such as age, sex and genetic predisposition^2^. Beyond acute coronary syndromes, CAD contributes to heart failure and arrhythmias, imposing a substantial economic burden.

Despite advances, diagnostic accuracy is still challenging: up to 70% of patients referred for coronary angiography show no flow-limiting stenosis despite ischaemic symptoms^3–5^. Many are now classified as ischaemia with non-obstructive coronary arteries (INOCA), a cohort with high morbidity and frequent rehospitalisation^6^. Microvascular dysfunction can also coexist with angiographically significant lesions (≥ 70%)^7^.

Current guidelines recommend estimating pre-test probability, then applying non-invasive imaging—coronary CT, stress echocardiography, myocardial perfusion imaging or stress MRI—before invasive angiography is considered^7,8^. Yet visual assessment of 40–80% stenoses is often inaccurate, particularly in multivessel disease, leading to functional misclassification and inappropriate therapy^9–11^.

In the past, several clinical studies have already demonstrated high sensitivity and specificity of magnetocardiography (MCG) in detecting ischemic myocardium both at rest and under stress^1–7^. In this context, a stress MCG consists of measurement under resting conditions and one after an appropriate ergometry (stress). There is also evidence suggesting that MCG is suitable for detecting microvascular dysfunction^8^.

An MCG system is a functional imaging system that measures the magnetic field generated by the activity of heart cells. This means that the electric current that causes the heart muscle cells to contract generates a weak bio-electromagnetic field. This magnetic field is present throughout the body and can be up to 5000 times stronger than that of the brain, but the magnetic field strength is clearly within the pico-Tesla range. This is the reason for using quantum sensing for an MCG, as even the earth’s magnetic field has a field strength in the µT range, cf. Figure 12.1 in Malmivuo, 1995^9^. Furthermore, shielding and/or other methods, e.g. filtering, are commonly applied to reduce interference to the sensitive MCG signal and enhance the signal-to-noise ratio. The advantage is that the sensors can be positioned directly over the human chest and the magnetic field can be measured directly from the source, the heart itself, and cannot be distorted by tissue or membranes.

However, detection and analysis of the heart’s magnetic field offer several important physical advantages over the ECG. With ECG it is only possible to measure cross membrane currents, as the cell membrane serves as an insulator for electric fields. Using MCG enables us to overcome these boundaries and measure intracellular currents, as their magnetic field is not shielded by the cell membrane. To extract this hidden information, we analyze specific parameters designed to quantify the total ion current—a methodological approach we term magnetoionography (MIG).

Unlike conventional MCG, whose parameters primarily reflect extracellular propagation currents, MIG is engineered to probe electrophysiological events at the sub‑cellular level. In basic research, such intracellular currents can only be visualized using fluorescent tracers or the patch‑clamp method, in which a glass micropipette forms a giga‑seal with an individual cardiomyocyte to record ionic currents directly.

By combining MIG with conventional MCG parameters, one obtains a two‑layer view of cardiac electrophysiology. MIG captures the trigger—sub‑cellular ion‑channel activity—while MCG depicts the downstream consequence, namely the extracellular propagation currents. This makes MIG particularly attractive for clinical and scientific applications to detect for example disturbances in calcium handling and repolarization heterogeneity and immediately observe how these perturbations translate into macroscopic conduction patterns.

This study investigates whether adding MIG-derived parameters to a conventional MCG scoring improves the non-invasive detection of CAD compared with MCG alone in a cohort of CAD patients and healthy controls.

## Methods

The MCG system (CS-MAG II, Biomagnetik Park GmbH) used in the study is equipped with a helium cooled sensor array composed of 64 super conducting interference device (SQUID) first order gradiometers. To mitigate noise interference, the system is implemented in a magnetic shielding room. The sensors measure the tangential component of the cardiac magnetic field at a sampling rate of 500 Hz. Each measurement consists of a rest phase as well as a stress phase which follows a period of exercise using an integrated ergometer. In our stress protocol, participants pedaled at a consistent cadence, with the workload incrementally increased in predefined stages. Throughout the exercise, continuous 12-lead electrocardiogram (ECG) monitoring was simultaneously performed. The test continued until participants reached 85% of their age-predicted maximum heart rate, exhibited limiting symptoms, or met other standard termination criteria. Following the exercise phase, participants entered a recovery period in which MCG monitoring was continued and assessed before returning to baseline values (also referred to as stress).

After signal acquisition, heartbeats are automatically detected for both rest and stress phases and subsequently averaged into a single composite signal representing a single heartbeat. Therefore, the individual periodic heartbeat cycles are registered in time and arithmetically averaged so that one representative heartbeat is acquired for each channel. The root-mean-square (RMS) of the average signals is then calculated across all *N* active channels and can defined as reference.

$$R\left( t \right): = \sqrt {\frac{1}{N}\mathop \sum \limits_{{n = 1}}^{N} B_{n}^{2} \left( t \right)}  $$

whereby $B_{n} (t)$ represents a single heartbeat that corresponds to the *n*^th^ sensor. Each sensor synchronously provides an individual heart signal that was recorded at a different location. Since the distance from heart to sensor is different, each sensor refers to a different heart arial. In other words, the RMS signal is a geometric average over all channels that represents the least common denominator or reference signal of one representative heartbeat. Finally, special MCG filtering methods are applied on the averaged (and RMS) signals to isolate the 0.3 Hz and 100 Hz frequency range as well as to eliminate baseline drift. Finally, the QRS-complex and T-wave are automatically segmented.

By focusing on the T-wave physiology, we first investigate conventional MCG parameters for characterizing CAD, which can also be applied to ECG diagnostics. The simplest of these parameters is the Heart Rate, calculated from both the rest and stress MCG measurements. In contrast to ECG signals, SQUID-based MCG measurements provide a higher signal-to-noise ratio and geometrical resolution, since sensor array is placed directly above the human chest plane. Furthermore, we transform a combination of four specific MCG parameters to a scoring system, the Total Score, that can be used for CAD detection.

Due to the nature of magnetic fields, an MCG is capable of measuring intracellular cardiac currents, which are isolated by cell membranes (during plateau phase of the action potential) and consequently hidden from electrocardiographic measurements. Therefore, we investigate specific MCG parameters that intend to quantify this intracellular cardiac current flow. These currents are responsible for a pathological pattern during repolarization, resulting from a disturbed interaction of intracellular and membranous ion fluxes. The functional imaging system including the analysis of these cellular currents is called magnetoionography (MIG).

### Magnetocardiography (MCG) analysis

**Fig. 1:**
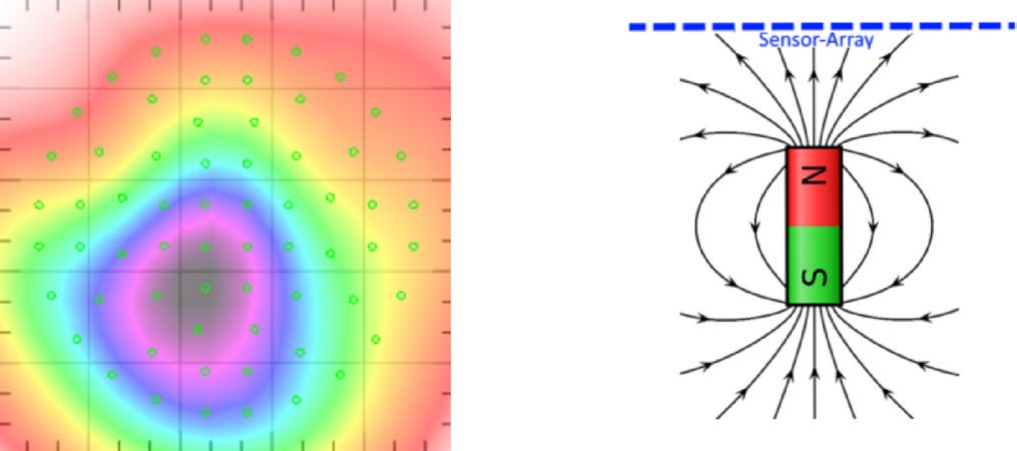
Monopolarity Index close to 1 by comparing the bio-magnetic activity with a bar magnet.

**Fig. 2:**
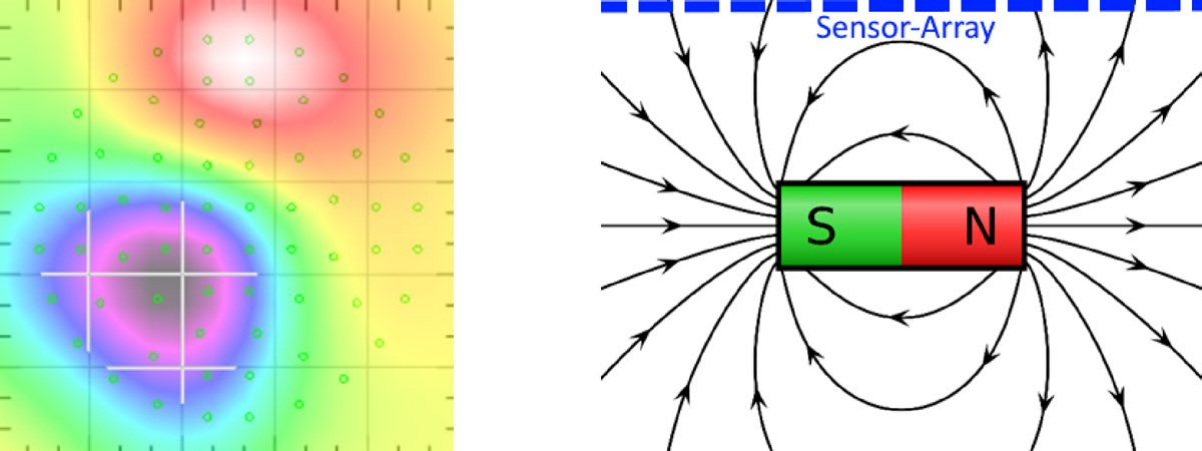
Dipolarity Index close to 1 by comparing the bio-magnetic activity with a bar magnet.

The orientation of the electrical current to the sensor array depends on the relative position of the heart itself and on the direction of the electrical current flow within the myocardial cells. The resulting magnetic field, this orientation is also on the Magnetic Field Map, which can be generated after various post-processing steps of the measured data. We can quantify the orientation of the electric flow by calculating the degree of monopolarity that the magnetic field appears to take in the magnetic field map. The Monopolarity Index is defined as the normalized sum of *N* sensor values $\:{B}_{n}$

$$ M: = \frac{{\left| {\mathop \sum \nolimits_{{n = 1}}^{N} B_{n} } \right|}}{{\mathop \sum \nolimits_{{n = 1}}^{N} \left| {B_{n} } \right|}}.$$

for a single sample, such that a dynamic Monopolarity Index is defined as *M(t)*, see Ehrlen et al.^10^. It quantifies the correlation between the characteristic of the magnetic field map to a non-dipole-like pattern, see Fig. 1. The Dipolarity Index is therefore defined as $\:D≔1-M$ or $\:D\left(t\right)≔1-M\left(t\right)$, respectively, see Fig. 2.

#### The total score

The Total Score is the amalgamation of several parameters which are sensitive to predicting CAD. These parameters are the STT-Analysis, T-(Wave-) Dispersion, Vector magnetocardiography (VMCG) and the so-called Park-Lam Phenomenon (PLP, named after the inventors). The specific combination of these four parameters by the means of the Total Score arithmetic is built off the article from Shin et al.^11^. The following is a brief overview of the basic theory. However, it should be emphasized that all parameters basically decode the electrophysiological characteristics via the ratio of the individual heart signals to the reference signal.

1. STT-Analysis is a geometrical analysis of the current dipole moment and the magnetic dipole pattern during the STT segment, see Shin et al.^11^. It consists of three components with an individual score:
  - Current Moment Dynamics: A classical parameter within the STT-Analysis, which should be highlighted, is defined as Current Moment Dynamics. It is calculated from the maximum slope of current dipole moment during the T-Wave. We observe the highest dynamic or velocity of electrical dipole moment that corresponds to maximal in- or decrease of potential energy released interior the excitation regression phase. The electrical dipole moment signal results from the inversion of the linearized Biot-Savarts’ modelling ansatz.
  - Pole Distance Dynamics: The second member within the STT-Analysis is the largest spatial distance between the maximum and the minimum of the magnetic dipole interior the magnetic field map during the STT segment.
  - Current Angle Dynamics: Last, but not least, we measure the rotational dynamic by the largest angle of the rotation axis of the magnetic dipole extrema interior the magnetic field map during the STT segment.

In other words, if the magnetic field of the heart fluctuates too inhomogeneously or is unbalanced, this parameter becomes pathologic.

**Fig. 3:**
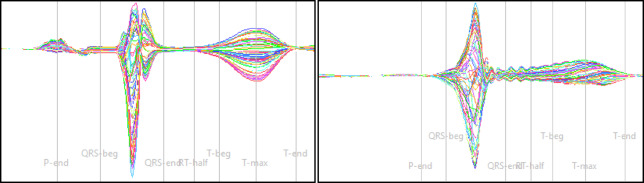
A healthy/homogenous case on the left-hand side, and a sick/inhomogenous case on the right-hand side.

2. T-Dispersion is the quantification of transmural repolarization dispersion, see Shin et al.^11^. In other words, if the magnetic fields of the individual channels are too dispersed at the peak time of the T-wave, this parameter becomes pathological, see the homogenous and inhomogeneous T-waves in Fig. 3.
3. VMCG quantifies the reconstructed trajectory of cardiac magnetic dipole moment wander. Coronary artery disease can be detected or analyzed by evaluating and scoring the distance between start and end point, since they have to be 0 in a continuum. As in mentioned in Current Moment Dynamics, the magnetic dipole moment has to calculated by solving the inverse problem, see Shin et al.^11^.
4. The PLP parameter is a quantification of the dipolar or non-dipolar (also defined as monopolar) pattern interior the magnetic field map, see Fig. 2. An upward or downward trend of the PLP result evaluates the proportion of the injury current inside the STT segment, see Park et al.^10^. Therefore, the Monopolarity Index is used for an appropriate quantification. In this article, “monopole” is used as an alternative wording for non-dipole and the Monopolarity Index is the normalized sum of all sensor values.

$$M_{n}  = \frac{{\left| {\sum B_{n} } \right|}}{{\sum \left| {B_{n} } \right|}} $$

5. It varies between 0 and 1, whereby 1 indicates a high imbalance (all sensors, i.e. for every index *n*, are either positive or negative). The so-called Dipolarity Index $\:{D}_{n}$ can be in turn defined as.

$$ D_{n} : = 1 - M_{n}  $$

After a normalized thresholding, the combination of these four parameters builds the Total Score, which has a range from 0 to 10 for a rest-only MCG measurement and from 0 to 20 if a stress measurement was also applied. The main parameter for detecting ischemia consists in STT-Analysis and T-Dispersion, whereby VMCG and PLP are implemented as enhancing parameters. This means that VMCG and PLP only take part in the sum if at least one of main parameters has at least one point. A pathologic finding is determined if the Total Score is at least two points.

Related to the Current Moment Dynamics is the Current Moment Increase parameter which represents the maximum convex slope of current dipole moment within the T-Wave, i.e. during $\:{T}_{beg}$ and $\:{T}_{Max}$. In order to get a quantitative value of the sum of electrical influences during the T-Wave the average of reference signal was defined as additional parameter.

### Magnetoionography (MIG) analysis

**Fig. 4:**
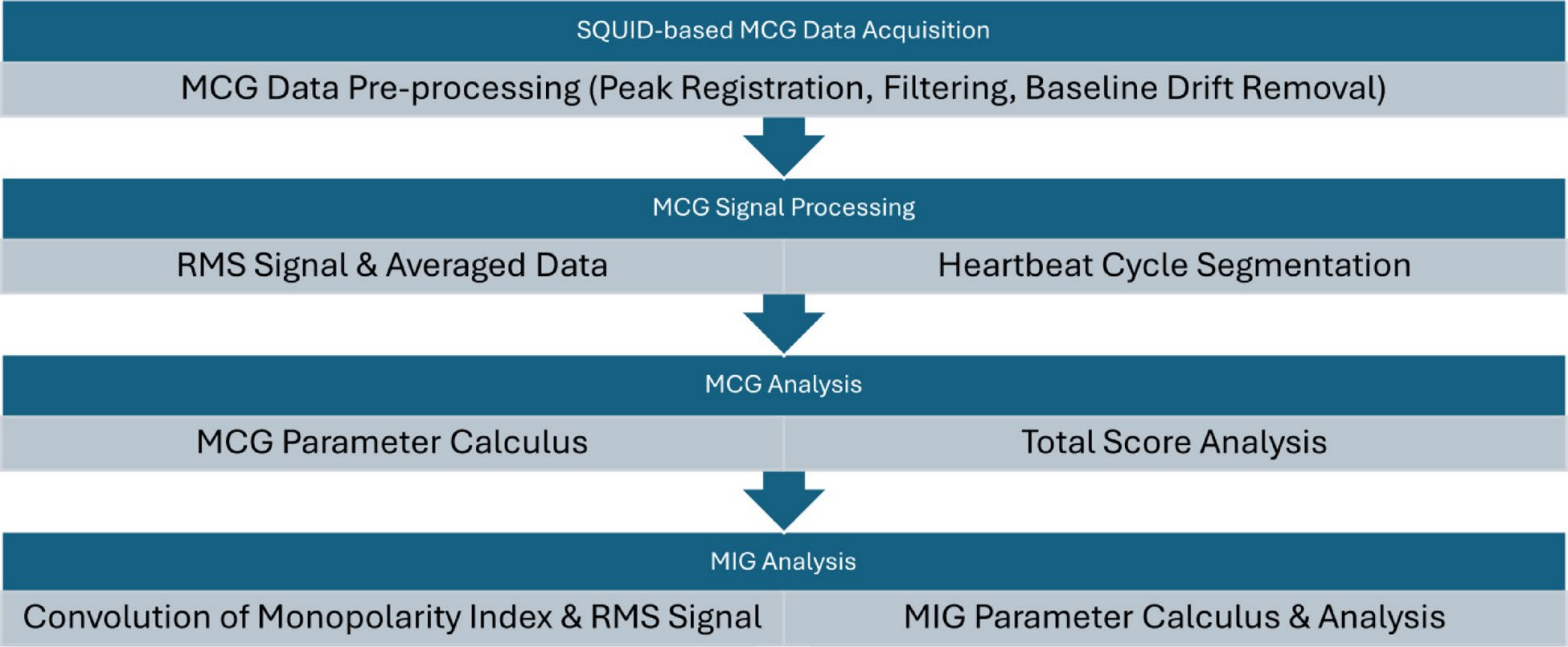
MCG signal processing for the combined CAD analysis pipeline.

MIG emerges as a new method for analyzing MCG data or signals. It results from the understanding that intra-cellular ion fluxes are isolated by the cell membrane and therefore the ECG is blind to these currents. However, the magnetic fields penetrate all tissue and are measured by the MCG. MIG is therefore an algorithmic extension to capture this entropy increase without requiring any changes to the measurement procedure, for more information refer to^12^.

To characterize the intracellular currents, we define the (dipolar) ionic flux function, *I(t)*. This function represents all directed currents that are responsible for the dipole characteristic within the magnetic field map. Further, we assume that *I(t)* is the result of convolving the Dipolarity Index $\:D\left(t\right):=1-\frac{1}{N}{\sum\:}_{n=1}^{N}{B}_{n}^{2}\left(t\right)$ with the reference signal *R(t)*.

The characteristic function *I(t)* is therefore given by

$$\:I\left(t\right):=R\left(t\right)\otimes\:D\left(t\right).$$.

We defined $\:{T}_{beg}$ as the highest upward slope during the STT segment or T-Wave and $\:{QRS}_{end}$ as the end of the QRS complex. During excitation regression, a non-pathological cell should empirically have directed ion exchange within this phase, forcing a dipolar magnetic field pattern, see Fig. 2. Due to the approach of characterizing dipolar currents by computing I(t), we calculate the arithmetic average of *I(t)* and the monopolar ionic flux function

$$\:J\left(t\right):=R\left(t\right)\otimes\:M\left(t\right)$$.

with $\:M\left(t\right):=\frac{1}{N}{\sum\:}_{n=1}^{N}{B}_{n}^{2}\left(t\right)$ (please refer to the PLP parameter) during various specific segments, i.e. $\:{QRS}_{end}$ to $\:{T}_{beg}$, and $\:{QRS}_{end}$ to $\:{T}_{End}$, or the QRS complex to quantify the influence or portion of monopolar (non-dipolar) ion fluxes to the reference signal *R(t)* within this segment, please see Fig. 4 for a flipchart of the MCG and MIG analysis pipeline. In other words, in addition to the RMS signal *R(t)*, we calculate two further reference signals *I(t)* and *J(t)* that reflect the pattern of the magnetic field map, where *I(t)* contains the dipole and *J(t)* the monopole influences. Thus, if *I(t)* dominants over *J(t)*,* the ionic fluxes is moving in axial direction*,* see* Fig. 2. A diffuse directed flux (*J(t)* is dominant), on the other hand, causes a monopole pattern within the magnetic field map, see Fig. 1. Quantification is performed in the first instance by calculating mean values. I.e. we calculate for the dipolar portion or index for the repolarization through to the T-Wave with the mean value of *I(t)* within the STT segment.

Further, the maximum convex slope dipolar ion flux function *I(t)* within $\:{QRS}_{end}$ and $\:{T}_{Max}$was computed to obtain an ion flux release parameter such as Current Moment Increase. Since the $\:{Ca}^{2+}$ions could be a crucial type of ion fluxes for this region, this parameter is defined as Calcium Release Velocity.

However, the introduced MIG parameters are based on characteristic function *I(t)* and can be quantified by using common analysis methods, like calculating the average, the slope, or the peaks over time. Furthermore, all given parameter for MCG or MIG are straight normalized such that they differ only on the variation of segmentation or isoline. Thus, by using automatic detection algorithms and standardized settings, such as the isoline at the beginning of the QRS complex, all analyses are completely reproducible, and a bias can be excluded. All analysis were carried out without prior knowledge of the coronary angiography findings.

### Patients

The CAD cohort consisted of individuals who underwent coronary angiography at Asklepios Hospital Hamburg-Harburg, Germany, with approval from the Ethics Committee of the Medical Association of Hamburg. Written informed consent was obtained from all participants. All methods were performed in accordance with the relevant guidelines and regulations. A definitive diagnosis of CAD was established based on the presence of at least 70% stenosis in at least one proximal epicardial coronary artery. MCG measurements were acquired prior to the performance of coronary angiography. All CAD patients included in the study exhibited stenosis in one or more coronary arteries.

The healthy volunteers in the study had to be free of clinically manifest diseases, not taking any medication, not have any somatic cardiovascular risk factors and not show any signs of acute infection.

Exclusion criteria for the patients were as follows:

- Rhythm disturbances (atrial fibrillation, atrial flutter, unsustained ventricular tachycardia).
- Bundle branch blocks.
- Previous MI.
- Pathological Q-wave.
- Echocardiographically determined regional wall motion abnormalities.
- Severe hypertrophy or dilation.
- MCG artefacts due to hardware malfunctions (confirmed by consensus of two experienced MCG readers).

Patients with implantable devices were also excluded due to strong magnetic disturbances.

## Results

This study included 93 participants with CAD (77 men, 16 women, mean age 70.0 +/- 12.2 years) and 36 healthy controls (30 men, 6 women, mean age 58.4 +/- 11.1 years, cf. Table 1).

**Table 1:** Baseline characteristics table (p-values regarding Chi^2^-test resp. Mann-Whitney).

	CAD (*n* = 93)	Control (*n* = 36)	*P*
Gender (♂)	77	30	n.s.
Age	69 ± 11	53 ± 10	< 0.001
Body mass index	28 ± 4	24 ± 2	< 0.001
Hypertension	84	0	< 0.001
Hyperlipidemia	65	0	< 0.001
Multivessel disease	53	0	< 0.001
Diabetes	14	0	< 0.05
Reduced Ejection Fraction (not normal)	68	0	< 0.001

**Fig. 5:**
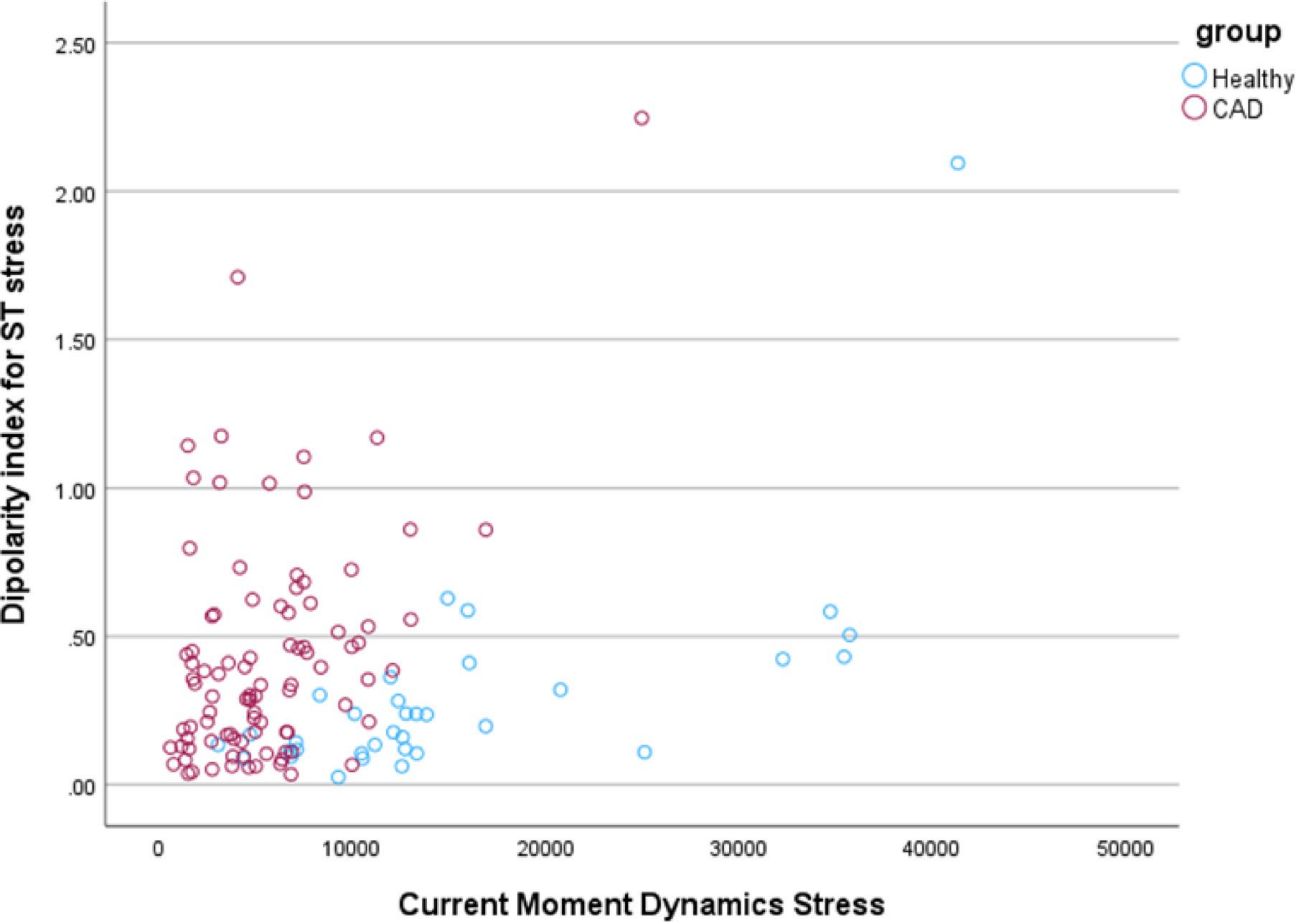
Visualization of the two best scoring parameters including newly developed **MIG** parameters. In combination with the parameter “Heart Rate Stress”, they achieved a sensitivity of 93.5% and a specificity of 85.3%, obtained with leave one out cross validation.

**Fig. 6:**
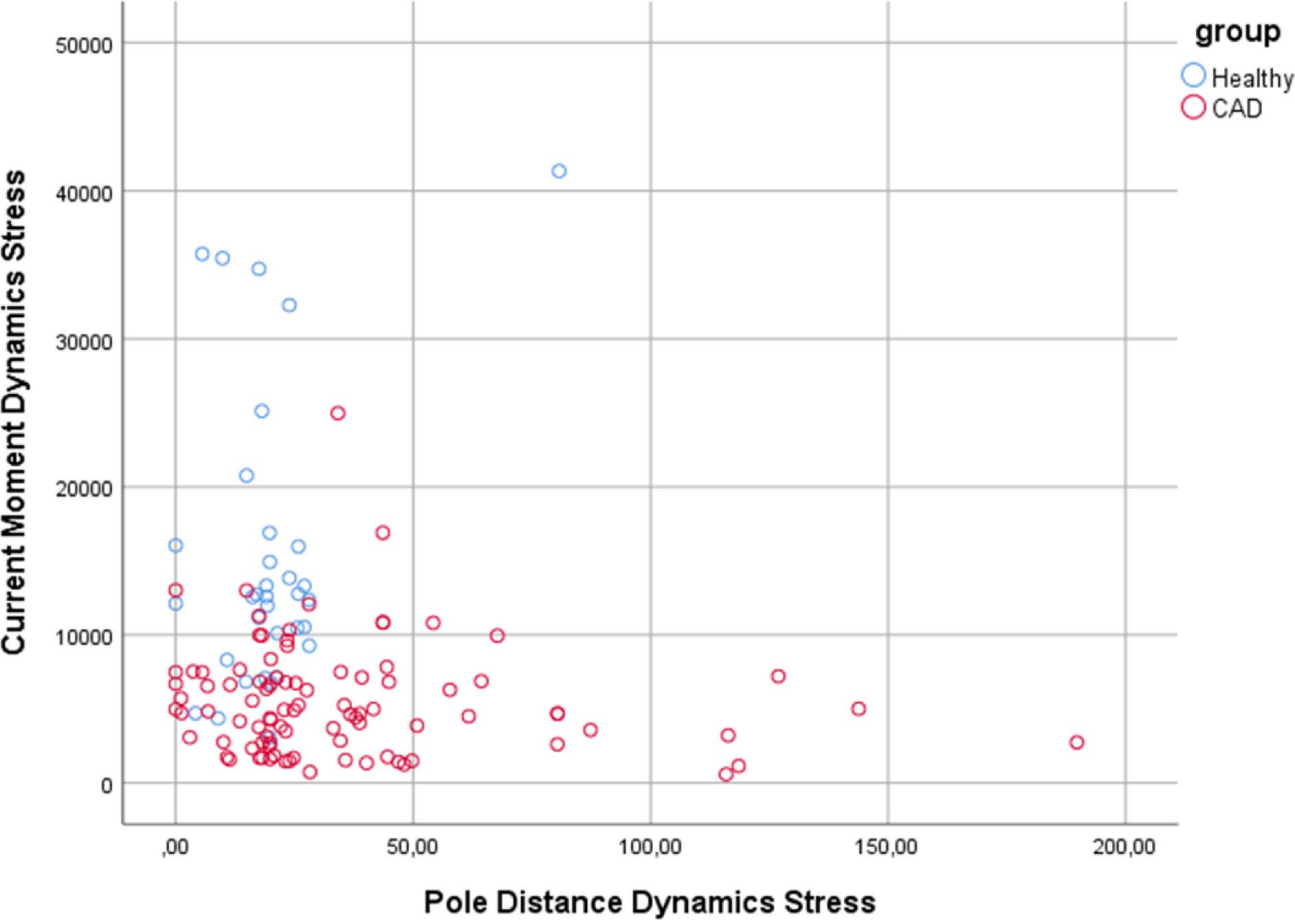
Visualization of the two best scoring classical **MCG** parameters. In combination with the parameter “Average RMS for ST Segment Rest” it achieved a sensitivity of 90.3% and specificity of 76.5%.

Stepwise linear discriminant analysis (LDA) was applied to select the best sets of features for MCG and MIG parameters (cf. Table 2). For classical MCG analysis, the three best parameters were found to be “Current Moment Dynamics Stress”, “Average RMS for ST Segment Rest” and “Pole Distance Dynamics Stress” yielding 90.3% sensitivity and 76.5% specificity with leave one out cross validation. Including MIG parameters in the analysis resulted in an increase to 93.5% sensitivity and 85.3% specificity, achieved by the combination of parameters “Heart Rate Stress”, “Current Moment Dynamics Stress” and “AverageDIxRmsQrsEndtoTbeg (ST)” (also defined as Dipolarity Index for ST Segment Stress). The Current Moment Dynamics parameter indicates the maximum (pseudo-) current increase during $\:{T}_{beg}$ and $\:{T}_{Max}$ or the maximum dynamic interior repolarization. Dipolarity Index for ST Segment indicates the average proportion of dipolar, i.e. axial and rectified, repolarization current that occurs during $\:{QRS}_{end}$ and $\:{T}_{beg}$. Compare Figs. 5 and 6 for visualization of separation.

**Fig. 7:**
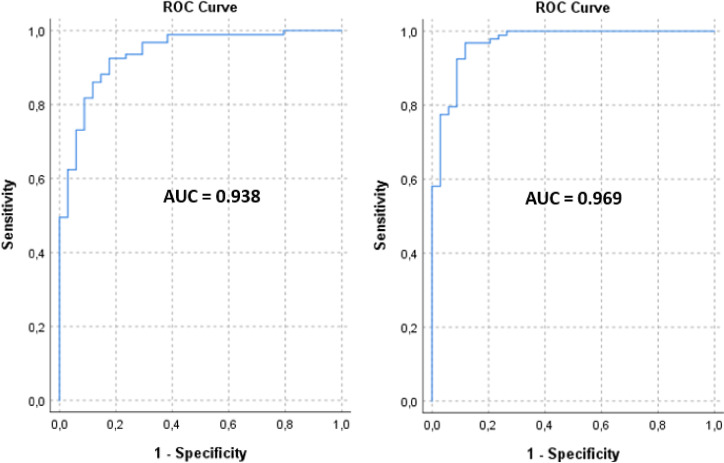
ROC curves left panel **MCG** parameters only (area under curve **AUC = 0.938**, 95% CI 0.911–0.985, *p* < 1E-13), right panel with **MIG** parameters (**AUC = 0.969**, 95% CI 0.938-1.0, *p* < 1E-15).

We used also alternative classification methods such as logistic regression and random forest. Logistic regression was not quite successful, whereas random forests (RF) received good classification results (ROC = 0.9, specificity = 0.74 and sensitivity = 0.85). However, best results were achieved by LDA (ROC = 0.97, cf. Figure 7). This method has several assumptions: (i) Multivariate Normality: Each class is assumed to have features that are normally. (ii) Equal Covariance Matrices: All classes share the same covariance matrix, implying. (iii) Linearity: The relationship between the features and the class labels is linear. In our analysis, we evaluated these assumptions as follows: (i) We assessed the distribution of each feature within classes using Q-Q plots. While some deviations from normality were observed, LDA is known to be relatively robust to moderate violations of this assumption. (ii) We employed Box’s M test to examine the equality of covariance matrices across classes. The test indicated no significant differences, supporting the assumption of homoscedasticity. (iii) Scatter plots and correlation analyses suggested a linear relationship between features and class labels, justifying the use of LDA. It is known from the literature that LDA still performs quite well even when the assumptions are violated. We used leave-one-out cross-validation to assess the generalizability of the model and to detect signs of overfitting. The results are virtually identical to the optimistic data set, including full groups.

**Table 2:** Basic statistics of calculated MCG/MIG parameters at stress and at rest as well as the corresponding p-values of the Mann-Whitney-U-test (Benjamini-Hochberg corrected).

	Stress			Rest		
Parameters (segment)	Healthy	CAD	P-value	Healthy	CAD	P-value
MCG – Parameters						
Heart Rate	103 ± 22	84.9 ± 19	**0.005**	69.7 ± 11	70.5 ± 14	n.s.
PoleDistanceDynamics (ST-T)	19.7 ± 13	35.4 ± 33	n.s.	18.5 ± 8.9	33.5 ± 34	n.s.
AverageRmsTbegtoTend (T-wave)	1.58 ± 0.81	0.89 ± 0.61	**7.3E-06**	1.43 ± 0.73	0.896 ± 0.63	**9.2E-05**
Tdispersion (T-wave)	6.25 ± 1.7	7.93 ± 2.6	**0.007**	6.54 ± 1.6	8.65 ± 2.7	**0.002**
ST-T Score	0.147 ± 0.36	0.699 ± 0.83	**0.03**	0.139 ± 0.42	0.591 ± 0.84	n.s.
PlpScore (ST-T)	0.5 ± 0.51	0.473 ± 0.5	n.s.	0.556 ± 0.5	0.441 ± 0.5	n.s.
VmcgTDistanceMagneticDipole (ST-T)	0.09 ± 0.05	0.11 ± 0.09	n.s.	0.11 ± 0.06	0.11 ± 0.08	n.s.
CurrentMomentDynamics (ST-T)	30.4 ± 19.6	11.1 ± 7.8	**5.5E-09**	20.6 ± 12.0	11.02 ± 8.0	**0.003**
CurrentMomentIncrease (ST-T)	1302 ± 780	544 ± 440	**1.9E-08**	770 ± 440	584 ± 720	**0.003**
AverageRmsTbegtoTend (T-wave)	1.58 ± 0.81	0.89 ± 0.61	**7.3E-06**	1.43 ± 0.73	0.896 ± 0.63	**9.2E-05**
TotalScore	0.676 ± 1.7	3.17 ± 2.5	**1.1E-05**	0.75 ± 1.4	3.18 ± 2.5	**1.1E-05**
MIG – Parameters						
CalciumReleaseVelocity (T-wave)	35.4 ± 18	16.2 ± 9.4	**5.9E-08**	25.6 ± 10	14.3 ± 7.9	**1.8E-07**
AverageMIxRmsQrsInterval (QRS)	1.35 ± 0.94	0.66 ± 1.3	**6.9E-05**	1.2 ± 0.66	0.58 ± 1.2	**3.0E-06**
Dipolarity index for ST	0.3 ± 0.36	0.43 ± 0.38	n.s.	0.22 ± 0.24	0.39 ± 0.39	n.s.
AverageMIxRmsTbegtoTend (T-wave)	0.425 ± 0.4	0.173 ± 0.35	**1.9E-05**	0.347 ± 0.33	0.204 ± 0.46	**0.0002**

In our study, we conducted multiple statistical tests, which inherently increases the risk of Type I errors (false positives). To mitigate this risk and control the false discovery rate, we applied the Benjamini-Hochberg procedure to adjust the p-values.

### Limitations

While our findings demonstrate a promising diagnostic potential of MIG in enhancing the accuracy of CAD detection, several limitations must be acknowledged to contextualize the results and guide future research. Our CAD cohort (mean ± SD age = 70 ± 12 y; 83% male) reflects the epidemiology of patients referred for coronary angiography at a tertiary German center but inevitably introduces age- and sex-related bias. Repolarization of heterogeneity and calcium kinetics change with advancing age. Therefore, MIG thresholds derived here may over- or under-estimate risk in younger adults. Female often show QTc intervals which are longer than in males, especially after puberty. This sex difference is well-documented and has clinical relevance, particularly in assessing the risk of drug-induced long QT syndrome and torsades de pointes.

The study cohort was predominantly composed of individuals from a relatively homogenous geographic and ethnic background, with patients enrolled from a single center. This limits the generalizability of our findings to broader, more diverse populations. Age, sex, and genetic variations are known to influence cardiac electrophysiological properties, and future studies must include multi-ethnic, gender-balanced (targeting ≥ 40% female representation), and age-diverse cohorts to ensure broader applicability of the diagnostic model. A further goal would be to validate MIG metrics in pediatric and adolescent populations, where intracellular calcium handling differs markedly.

Furthermore, the single-center nature of the investigation itself poses a risk of site-specific bias in data acquisition, patient management, and clinical decision-making. Therefore, multicenter studies are essential to validate the reproducibility and robustness of MIG-derived parameters. With 93 CAD patients and 36 healthy controls, the sample size—while sufficient to establish proof-of-concept—remains limited for drawing definitive conclusions. Additionally, the exclusion criteria may inadvertently limit the clinical scope of the technique. Broader inclusion criteria in future studies will be important for assessing MIG’s utility in real-world, heterogeneous patient populations.

The implementation of stringent criteria for selecting healthy volunteers should primarily aim at ensuring participant safety and achieving internal validity in clinical studies. It is important to acknowledge that such rigorous selection may limit the generalizability of the study results. Healthy volunteers often represent a subset of the population with specific characteristics—such as younger age, lower weight, absence of comorbidities (cf. clinical characteristics Table 1), and higher health literacy—that may not reflect the broader, more diverse population undergoing assessment for CAD. Therefore, in the future a more diverse participant pool that mirrors the heterogeneity of patients encountered in routine clinical practice should be considered.

This study focused solely on the diagnostic utility of MIG at a single time point. Longitudinal follow-up studies could provide valuable insights into the predictive power of MIG and MCG findings. Despite its potential, this technique requires highly sensitive SQUID sensors and a magnetically shielded room—resources not yet widely available in routine clinical practice. Despite its diagnostic promise, the implementation of MCG into everyday clinical practice is currently limited by several practical barriers. Addressing these challenges is essential to facilitate broader adoption and ensure clinical viability.

SQUID sensors require cryogenic cooling—typically with liquid helium—and magnetically shielded rooms to function optimally. These setups are expensive to install and maintain at the moment and are typically only available in highly specialized centers. The interpretation of MIG or MCG require a high level of expertise in biophysics, signal processing, and cardiac electrophysiology. There are currently no guidelines or protocols for MCG signal acquisition, preprocessing, segmentation, and analysis. This variability undermines reproducibility and comparability between centers and devices. To enable widespread use, standardized procedures and validated analysis pipelines must be developed and automated evaluation must be implemented. For MIG to be useful in routine diagnostics, an MCG system must be integrated into existing diagnostic pathways. However, MIG does not require any additional accessories or special hardware components to MCG, as it is a methodical extension. Application to systems with less stringent boundary conditions, e.g. with room temperature sensors, also needs to be explored.

## Discussion

This study presents a comprehensive analysis comparing the efficacy of MIG against traditional MCG, whereby MIG is an algorithmic extension and needs no additional equipment, only a software update. An application of MIG analysis into a clinical routine requires an integration of an MCG system and is, thus, limited to equal conditions. Our results indicate that MIG, with its potential capability to measure intracellular ion currents, provides superior sensitivity and specificity (93.5% and 85.3% respectively) in detecting CAD compared to previous studies conducted with traditional MCG and other diagnostic methods.

The significant differentiation between CAD patients and healthy controls using MIG underscores MCG’s potential as a non-invasive, risk-free diagnostic technique. Unlike traditional methods that predominantly focus on extracellular electrical activity, MIG delves deeper into cellular-level functions, providing insights into the molecular pathways that underpin heart function. This shift towards cellular-level analysis could revolutionize our approach to diagnosing CAD, offering earlier detection and more precise risk stratification.

As discussed in the introduction ischemic heart disease is a major global health issue and precise diagnosis still present a challenge in many cases. Non-invasive imaging tools such as CT cannot determine directly the functional relevance of a stenosis. Pathological findings often require subsequent invasive coronary angiography. Non-invasive methods are not suitable for diagnosing coronary microvascular dysfunction. The mentioned invasive methods always involve radiation exposure, contrast agent administration, procedural risk (a pressure wire must be placed in the coronary vessel), and high costs to the healthcare system. These investigations demand high expertise and clinical infrastructure and are usually only offered in specialized centers. The procedures are time- and cost-intensive and thus cannot always be implemented consistently in routine clinical practice.

Currently, no non-invasive diagnostic method exists that allows for direct assessment of coronary microcirculation in humans. Therefore, its evaluation relies on the measurement of invasively obtained parameters that reflect its functional status, such as myocardial blood flow and coronary flow reserve. MCG could offer a precise and complication-free diagnostic alternative in this context. MCG is non-invasive and requires neither radiation exposure nor the use of contrast agents. There are no specific side effects to be expected. The examination time ranges from just 1 min to approximately 15 min. Evaluation can be carried out automatically. This would constitute an excellent diagnostic tool not only for clinical settings but also for outpatient cardiology practices.

Comparatively, previous studies such as those shown in Table 3 have reported lower sensitivity and specificity rates. This discrepancy can be attributed to the advancements in MIG technology and methodology, emphasizing the evolution of cardiac diagnostic tools over time. The sum of sensitivity and specificity of our study is 178.8, which is marginally better than Gapelyuk et al. 2010, who achieved a sum of 176.0. They achieved an increase compared to the methodology used in Gapelyuk et al. 2007, by applying Kullback-Leibler entropy to field map topology as a discriminatory parameter. A future study will therefore also apply this methodology to our data, which we hypothesize will lead to further increases in sensitivity and specificity.

Recent work underscores the value of advanced analytic techniques. Askin et al. and Wang et al.^13,14^ showed that machine-learning–based “artificial algorithms” outperform conventional statistical models in predicting obstructive CAD, illustrating how data-driven approaches can sharpen diagnostic accuracy. Our findings complement this trend from a different angle: rather than refining risk prediction purely through algorithmic advances, we enhance the *underlying signal* by capturing intracellular ionic currents with magnetoionography. Together, the AI-driven improvements documented by Askin et al. and the physiologically enriched signal presented here suggest a future in which optimized analytics and novel biomagnetic markers are integrated to further reduce misclassification and unnecessary invasive testing. As mentioned in the limitations section, future work is explicitly called for multicenter, multinational studies with ≥ 40% female participants, validation in younger, pediatric, and ethnically diverse populations, head-to-head comparisons with guideline-matched imaging and longitudinal follow-up studies, automated analysis pipelines, and standardization initiatives.

In conclusion, in this single-centre study, the addition of MIG parameters improved the sensitivity and specificity of magnetocardiography for detecting CAD. While these findings demonstrate technical feasibility and indicate potential diagnostic value, they are derived from a relatively small, demographically skewed cohort and lack direct comparison with standard non-invasive tests. Larger, multi-centre studies—including head-to-head evaluations against guideline-recommended stress imaging—are required to fully assess the clinical utility of MIG. Those trials should build on established MCG protocols and systematically incorporate MIG into analysis workflows by software updates. In addition, MIG should be explored in broader cardiology settings—such as microvascular dysfunction, arrhythmias, and heart-failure phenotyping—and potentially in related biomedical fields, to define its diagnostic and prognostic value comprehensively.

**Table 3:** Overview of previous MCG studies on CAD and results. IHD = Ischemic heart disease, cmr = cardiac magnetic resonance Imaging.

Study	Year	Sensitivity	Specificity	Number of Patients
Van Leeuwen et al.^15^	2003	74%	80%	23 CAD without MI
Hailer et al.^16^	2005	73.3%	71.3%	177 CAD without MI, normal ECG findings
Park et al.^17^	2005	86.4%	82.5%	185 Patients with acute chest pain and no ST elevation
Tolstrup et al.^4^	2006	76.4%	74.3%	125 patients with presumed ischemic chest pain. All patients were chest pain free at the time of scanning
Gapelyuk et al.^18^	2007	84%	83%	101 CAD without MI
Kwon et al.^19^	2010	84%	85%	364 patients with the suspected acute coronary syndrome without ST segment elevation
Gapelyuk et al.^20^	2010	88%	88%	101 CAD without MI, reanalysis including Kullback-Leibler entropy
Chen et al.^21^	2014	86.7%	73.8%	15 CAD without MI
Wang et al.^14^	2024	96%	90%	1241 CAD (Train) and 58 (Validation) CMR + AI study; AUC of 0.997.
Tao et al.^22^	2025	83.8%	85.6%	1021 IHD (Train) and 150 (Validation), Without other cardiac defects and myocardial disease. AI Analysis
**Current study**	**2025**	**93.5%**	**85.3%**	**93 CAD without MI**,** analysis with Magnetoionography**

## Data Availability

The data that support the findings of this study are available from the corresponding author upon reasonable request.
